# ChatGPT’s diagnostic performance based on textual vs. visual information compared to radiologists’ diagnostic performance in musculoskeletal radiology

**DOI:** 10.1007/s00330-024-10902-5

**Published:** 2024-07-12

**Authors:** Daisuke Horiuchi, Hiroyuki Tatekawa, Tatsushi Oura, Taro Shimono, Shannon L. Walston, Hirotaka Takita, Shu Matsushita, Yasuhito Mitsuyama, Yukio Miki, Daiju Ueda

**Affiliations:** 1 Department of Diagnostic and Interventional Radiology, Graduate School of Medicine, Osaka Metropolitan University, Osaka, Japan; 2 Department of Artificial Intelligence, Graduate School of Medicine, Osaka Metropolitan University, Osaka, Japan

**Keywords:** Artificial intelligence, Natural language processing, Radiology

## Abstract

**Objectives:**

To compare the diagnostic accuracy of Generative Pre-trained Transformer (GPT)-4-based ChatGPT, GPT-4 with vision (GPT-4V) based ChatGPT, and radiologists in musculoskeletal radiology.

**Materials and methods:**

We included 106 “Test Yourself” cases from *Skeletal Radiology* between January 2014 and September 2023. We input the medical history and imaging findings into GPT-4-based ChatGPT and the medical history and images into GPT-4V-based ChatGPT, then both generated a diagnosis for each case. Two radiologists (a radiology resident and a board-certified radiologist) independently provided diagnoses for all cases. The diagnostic accuracy rates were determined based on the published ground truth. Chi-square tests were performed to compare the diagnostic accuracy of GPT-4-based ChatGPT, GPT-4V-based ChatGPT, and radiologists.

**Results:**

GPT-4-based ChatGPT significantly outperformed GPT-4V-based ChatGPT (*p* < 0.001) with accuracy rates of 43% (46/106) and 8% (9/106), respectively. The radiology resident and the board-certified radiologist achieved accuracy rates of 41% (43/106) and 53% (56/106). The diagnostic accuracy of GPT-4-based ChatGPT was comparable to that of the radiology resident, but was lower than that of the board-certified radiologist although the differences were not significant (*p* = 0.78 and 0.22, respectively). The diagnostic accuracy of GPT-4V-based ChatGPT was significantly lower than those of both radiologists (*p* < 0.001 and < 0.001, respectively).

**Conclusion:**

GPT-4-based ChatGPT demonstrated significantly higher diagnostic accuracy than GPT-4V-based ChatGPT. While GPT-4-based ChatGPT’s diagnostic performance was comparable to radiology residents, it did not reach the performance level of board-certified radiologists in musculoskeletal radiology.

**Clinical relevance statement:**

GPT-4-based ChatGPT outperformed GPT-4V-based ChatGPT and was comparable to radiology residents, but it did not reach the level of board-certified radiologists in musculoskeletal radiology. Radiologists should comprehend ChatGPT’s current performance as a diagnostic tool for optimal utilization.

**Key Points:**

- *This study compared the diagnostic performance of GPT-4-based ChatGPT, GPT-4V-based ChatGPT, and radiologists in musculoskeletal radiology.*
- *GPT-4-based ChatGPT was comparable to radiology residents, but did not reach the level of board-certified radiologists.*
- *When utilizing ChatGPT, it is crucial to input appropriate descriptions of imaging findings rather than the images.*

**Graphical Abstract:**

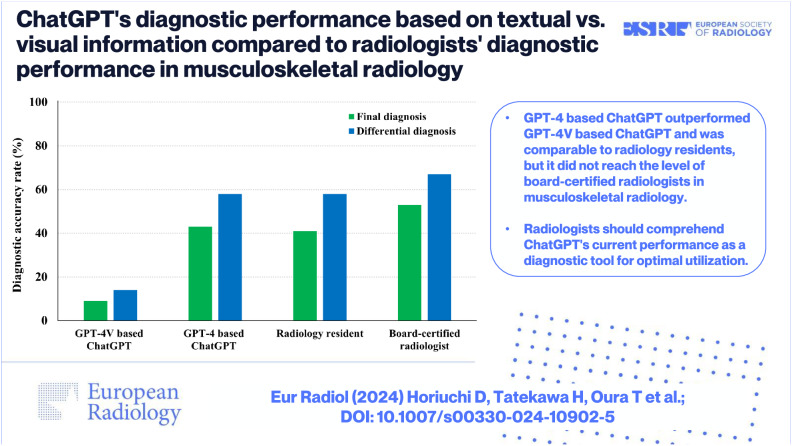

## Introduction

Chat Generative Pre-trained Transformer (ChatGPT) is a novel language model based on GPT-4 architecture, which demonstrates an impressive capability for understanding and generating natural responses on various topics [1–3]. Experts in various industries have been exploring the potential applications of ChatGPT and considering how its integration could improve efficiency and decision-making processes [4]. Furthermore, the recent GPT-4 with vision (GPT-4V) enables the analysis of image inputs and offers the possibility of expanding the impact of large language models [5]. Given the potential impact of ChatGPT in the medical field, healthcare professionals need to understand its performance, strengths, and limitations for optimal utilization.

Artificial intelligence has demonstrated notable benefits in the field of radiology [6, 7], and it also holds promise for improving diagnostic accuracy and patient outcomes in musculoskeletal radiology [8, 9]. ChatGPT has the potential to be a valuable tool in improving diagnostic accuracy and patient outcomes, and there have been some initial applications of ChatGPT in radiology [10–17]. GPT-3.5-based ChatGPT nearly passed a text-based radiology examination without any specific radiology training, and then GPT-4-based ChatGPT passed the examination [18, 19]. In musculoskeletal radiology, there has been only one study of ChatGPT, which focused on generating research articles [20].

Previous studies have evaluated the diagnostic performance of GPT-4-based ChatGPT from the patient’s medical history and imaging findings in the field of radiology [14, 17]. However, it remains unclear how ChatGPT’s diagnostic accuracy compares when using the images themselves (GPT-4V-based ChatGPT) or the written descriptions of imaging findings (GPT-4-based ChatGPT). Additionally, the comparison of diagnostic performance among GPT-4-based ChatGPT, GPT-4V-based ChatGPT, and radiologists has not been investigated. Current data are insufficient to determine whether the integration of ChatGPT into musculoskeletal radiology practice has the potential to improve diagnostic accuracy and reduce diagnostic errors.

The journal *Skeletal Radiology* presents diagnostic cases as “Test Yourself” to allow readers to assess their diagnostic skills. These diagnostic cases offer a means to evaluate the diagnostic performance of ChatGPT in musculoskeletal radiology and obtain insights into its potential as a diagnostic tool.

This study aimed to compare the diagnostic accuracy among GPT-4-based ChatGPT, GPT-4V-based ChatGPT, and radiologists in musculoskeletal radiology using the “Test Yourself” cases published in *Skeletal Radiology*.

## Materials and methods

### Study design

This study was approved by the institutional review board of our institution, and informed consent was not required since this study utilized only published cases. We input the patient’s medical history and descriptions of imaging findings associated with each case into GPT-4-based ChatGPT, and input the patient’s medical history and images themselves associated with each case into GPT-4V-based ChatGPT. Each ChatGPT generated the differential and final diagnoses, and we estimated the diagnostic accuracy rate of the outputs. Additionally, radiologists independently reviewed all the cases based on the patient’s medical history and images, and their diagnostic accuracy rates were evaluated. We then compared the diagnostic accuracy rates for the final diagnosis and differential diagnoses among GPT-4-based ChatGPT, GPT-4V-based ChatGPT, and radiologists. This study was designed according to the Standards for Reporting Diagnostic Accuracy Studies statement [21].

### Data collection

**Fig. 1:**
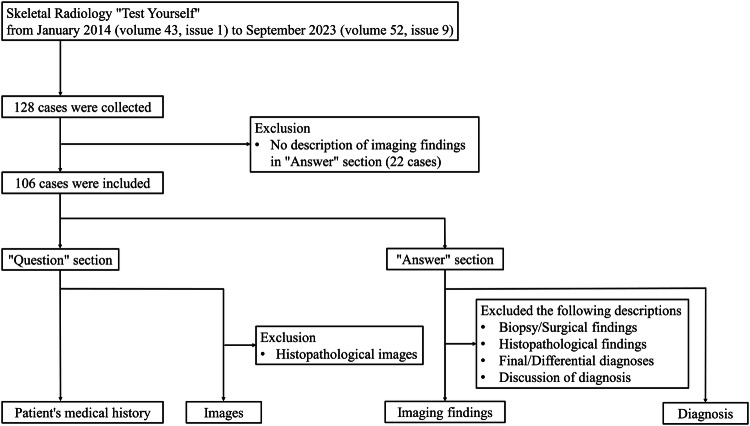
Data collection flowchart

The journal *Skeletal Radiology* publishes diagnostic cases in the “Test Yourself” section. We collected 128 consecutive “Test Yourself” cases from January 2014 (volume 43, issue 1) to September 2023 (volume 52, issue 9). We excluded 22 cases due to a lack of imaging findings text in the presented cases, and ultimately a total of 106 cases were included in this study. Each patient’s medical history and images (excluding pathological images) were collected from the “Question” section, and the descriptions of imaging findings were collected from the “Answer” section of each published case. The “Answer” section contained descriptions of biopsy/surgical findings, histopathological findings, final/differential diagnoses, and discussion of diagnosis; thus, we excluded these descriptions from imaging findings. The data collection flowchart is presented in Fig. 1.

### Input and output procedure for ChatGPT

**Fig. 2:**
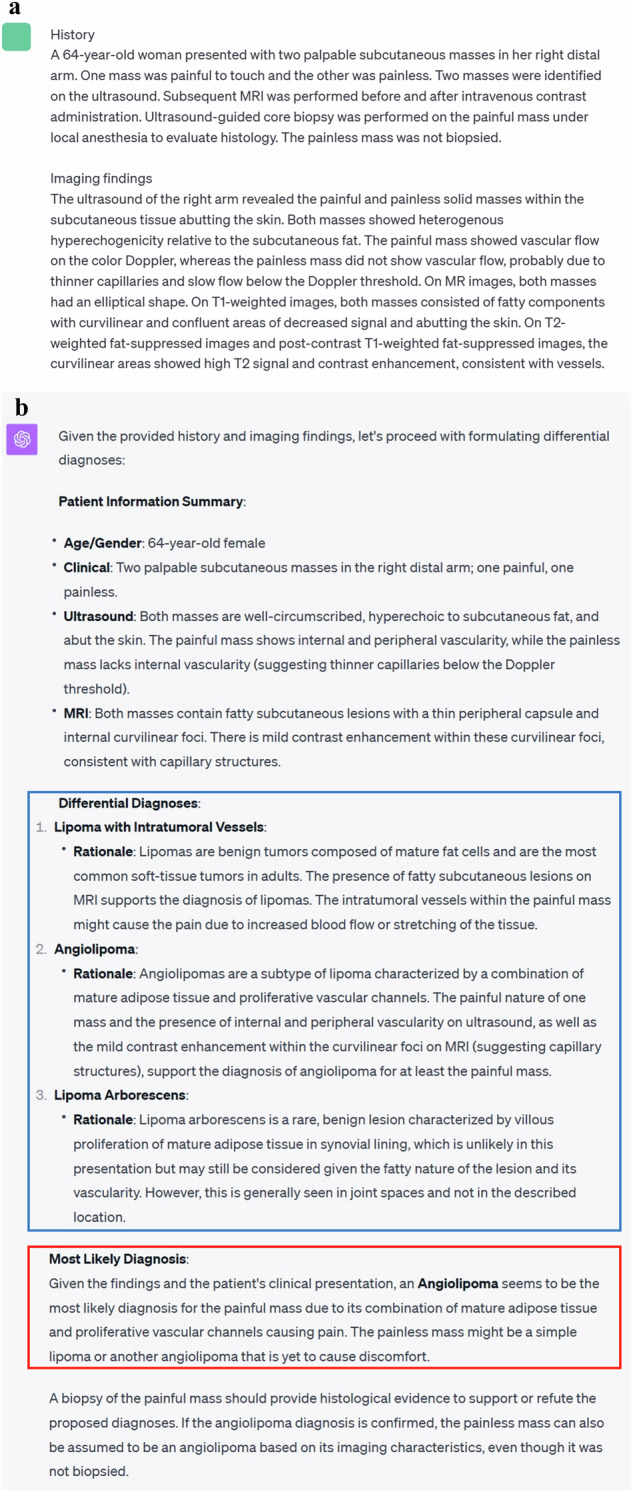
Input (patient’s medical history and imaging findings) and output examples of GPT-4-based ChatGPT. **a** Input texts to ChatGPT. **b** Output texts generated by ChatGPT. The differential diagnoses are outlined in blue and the final diagnosis is outlined in red. The final diagnosis generated by ChatGPT is correct in this case [33, 34]

**Fig. 3:**
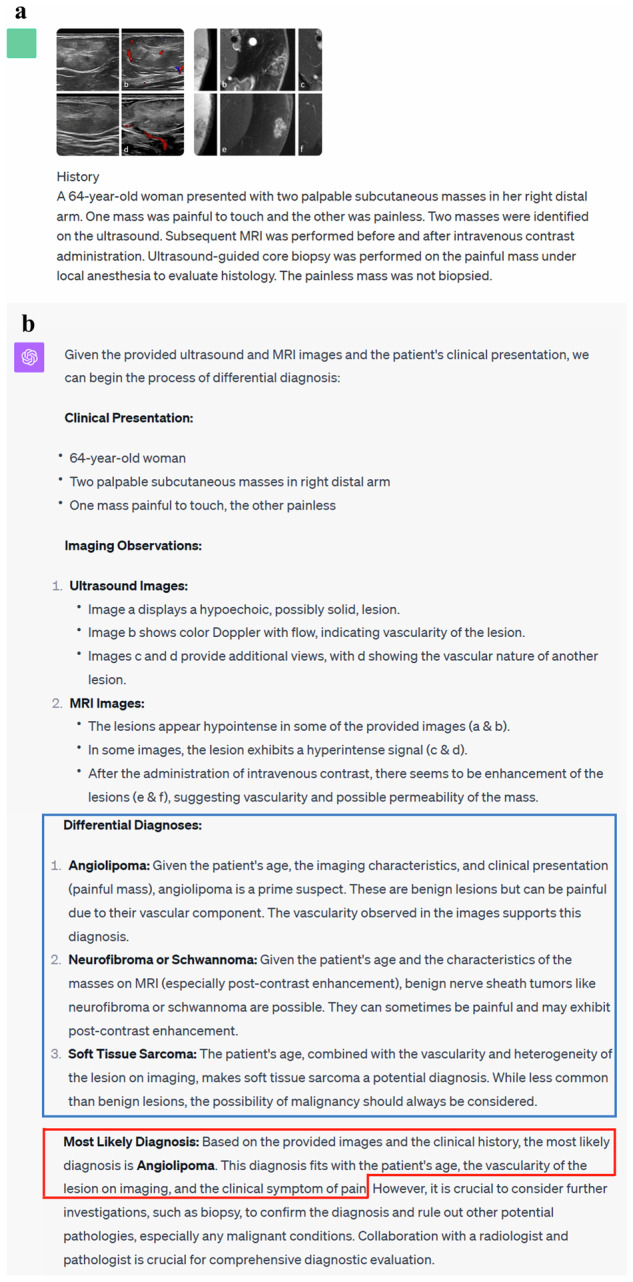
Input (patient’s medical history and images) and output examples of GPT-4V-based ChatGPT. **a** Input to ChatGPT. **b** Output texts generated by ChatGPT. The differential diagnoses are outlined in blue and the final diagnosis is outlined in red. The final diagnosis generated by ChatGPT is correct in this case [33, 34]

**Fig. 4:**
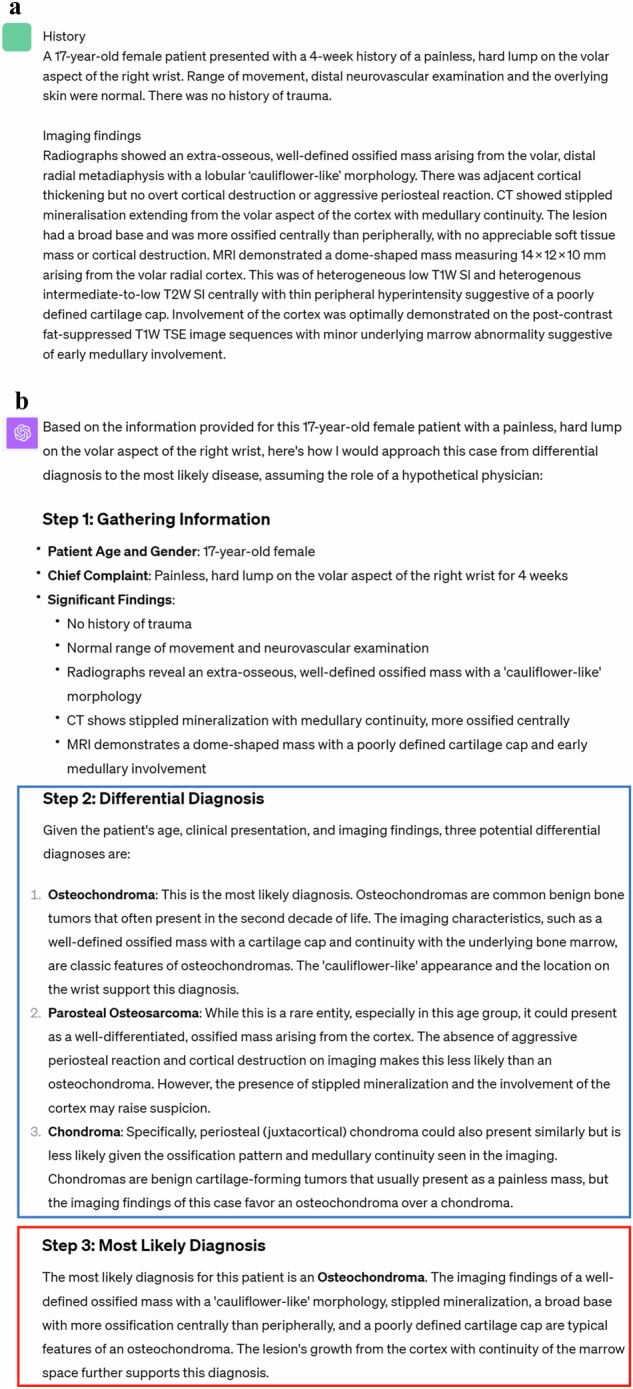
A challenging case example for GPT-4-based ChatGPT. **a** Input texts (patient’s medical history and imaging findings) to ChatGPT. **b** Output texts generated by ChatGPT. The differential diagnoses are outlined in blue and the final diagnosis is outlined in red. While the differential diagnoses generated by ChatGPT include the correct diagnosis, the final diagnosis is incorrect in this case (true diagnosis: parosteal osteosarcoma) [35, 36]

**Fig. 5:**
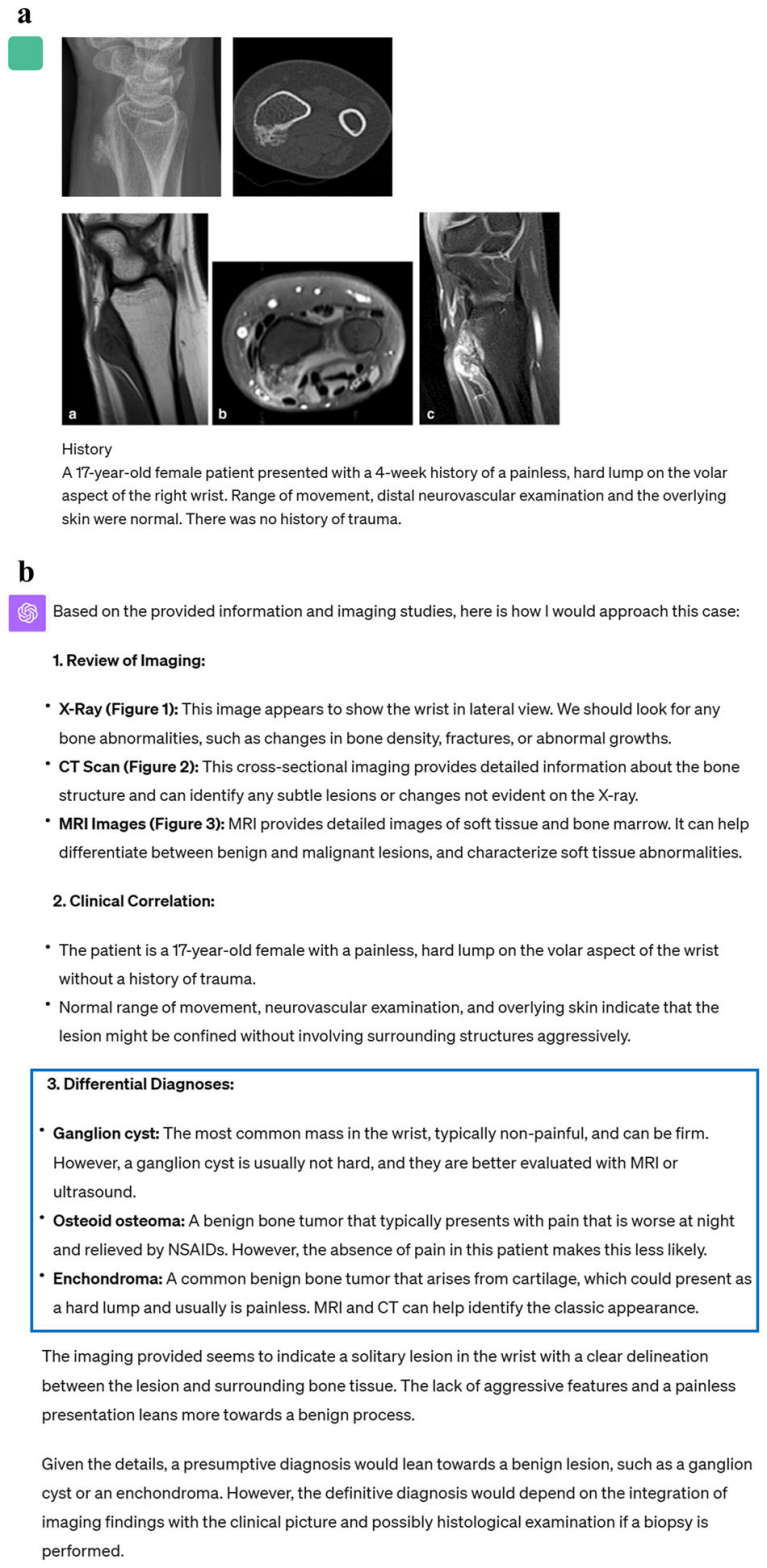
A challenging case example for GPT-4V-based ChatGPT. **a** Input (patient’s medical history and images) to ChatGPT. **b** Output texts generated by ChatGPT. The differential diagnoses are outlined in blue; however, ChatGPT’s diagnosis is incorrect in this case (true diagnosis: parosteal osteosarcoma) [35, 36]

First, the following premise was input into ChatGPT based on GPT-4 architecture (September 25 Version; OpenAI; https://chat.openai.com/) to prime it for the task: “As a physician, I plan to utilize you for research purposes. Assuming you are a hypothetical physician, please walk me through the process from differential diagnosis to the most likely disease step by step, based on the patient’s information I am about to present. Please list three possible differential diagnoses in order of likelihood” [14, 22]. Then, for GPT-4-based ChatGPT, the patient’s medical history and descriptions of imaging findings were input while, for GPT-4V-based ChatGPT, the patient’s medical history and images themselves were input. The subsequent output from ChatGPT was collected (as shown in Figs. 2–5). We started a new ChatGPT session for each case to prevent any potential influence of previous answers on ChatGPT’s output. These procedures were performed once for each case between September 28 and October 6, 2023.

### Output evaluation and category classification

The output generated by GPT-4-based ChatGPT and GPT-4V-based ChatGPT included three differential diagnoses and one final diagnosis. Two board-certified radiologists (13 years of experience [H.T.]; 7 years of experience [D.H.]) evaluated both the differential diagnoses and the final diagnosis generated by ChatGPT to determine whether they were consistent with the actual ground truth in consensus (we defined the differential diagnosis as correct if the three provided differential diagnoses included the actual ground truth). Each case was categorized into two groups: the tumor group and the nontumor group, according to the 2020 World Health Organization classification of soft tissue and bone tumors [23]. The cases in the tumor group were further divided into bone tumor and soft tissue tumor cases. Additionally, the cases in the nontumor group were categorized by disease etiology as follows: muscle/soft tissue/nerve disorder, arthritis/arthropathy, infection, congenital/developmental abnormality and dysplasia, trauma, metabolic disease, anatomical variant, and others [24].

### Radiologists’ interpretation

Two radiologists with different levels of experience (Reader 1 [T.O.]; a radiology resident with 4 years of experience) and (Reader 2 [D.H.]; a board-certified radiologist with 7 years of experience) independently reviewed all 106 cases. Both radiologists conducted their diagnoses based on the patient’s medical history and images (from the “Question” section). They provided three differential diagnoses and chose one as the final diagnosis for each case, and the diagnostic accuracy rates were evaluated. Both radiologists were blinded to the actual ground truth, as well as the differential and final diagnoses generated by ChatGPT.

### Radiologists’ interpretation with ChatGPT’s assistance

Following the initial interpretation, both radiologists independently reviewed all cases again, referencing the differential and final diagnoses generated by GPT-4-based ChatGPT and GPT-4V-based ChatGPT, respectively. They provided three differential diagnoses and chose one as the final diagnosis for each case, and the diagnostic accuracy rates with ChatGPT’s assistance were evaluated. Both radiologists were blinded to the actual ground truth.

### Statistical analysis

Statistical analyses were performed using R software (version 4.0.2, 2020; R Foundation for Statistical Computing; http://www.r-project.org/). Chi-square tests were conducted to compare the final and differential diagnostic accuracy rates between GPT-4-based ChatGPT and GPT-4V-based ChatGPT. Chi-square tests were also conducted to compare the final and differential diagnostic accuracy rates between GPT-4-based ChatGPT and each radiologist, as well as between GPT-4V-based ChatGPT and each radiologist. Furthermore, ChatGPT’s final and differential diagnostic accuracy rates for 1) the tumor and nontumor groups, and 2) the bone tumor and soft tissue tumor cases were compared with pairwise Fisher’s exact tests. Adjustment for multiplicity was not performed because this was an exploratory study. *p* < 0.05 was considered statistically significant.

## Results

### ChatGPT’s diagnostic accuracy: GPT-4-based ChatGPT vs GPT-4V-based ChatGPT

In all 106 cases, GPT-4-based ChatGPT (based on the patient’s medical history and imaging findings) and GPT-4V-based ChatGPT (based on the patient’s medical history and images) successfully generated three differential diagnoses and provided one final diagnosis. GPT-4-based ChatGPT’s diagnostic accuracy rates for the final and differential diagnoses were 43% (46/106) and 58% (62/106), respectively. In contrast, GPT-4V-based ChatGPT’s diagnostic accuracy rates for the final and differential diagnoses were 8% (9/106) and 14% (15/106), respectively. Both the final and differential diagnostic accuracy rates were significantly higher for GPT-4-based ChatGPT compared to GPT-4V-based ChatGPT (*p* < 0.001 and < 0.001, respectively).

### Comparison of the diagnostic accuracy between ChatGPT and radiologists

Regarding the radiologists’ diagnostic accuracy, Reader 1 (a radiology resident) achieved a final diagnostic accuracy of 41% (43/106) and a differential diagnostic accuracy of 58% (61/106). Reader 2 (a board-certified radiologist) achieved a final diagnostic accuracy of 53% (56/106) and a differential diagnostic accuracy of 67% (71/106).

**Fig. 6:**
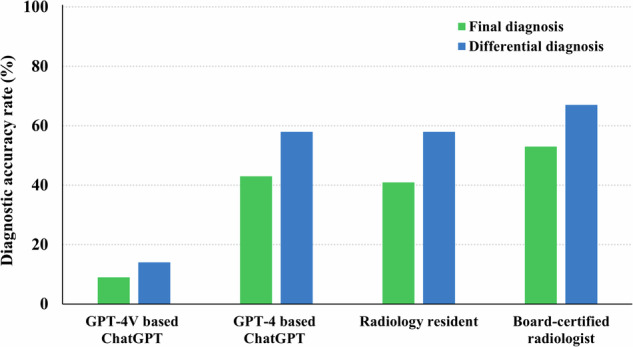
Diagnostic accuracy of GPT-4-based ChatGPT, GPT-4V-based ChatGPT, and radiologists

GPT-4-based ChatGPT’s diagnostic accuracy rates for the final and differential diagnoses were comparable and not statistically significantly different from those of Reader 1 (*p* = 0.78 and 0.99, respectively), but lower than those of Reader 2, though not significantly (*p* = 0.22 and 0.26, respectively) (Table 1) (Fig. 6). In contrast, GPT-4V-based ChatGPT’s diagnostic accuracy rates for the final and differential diagnoses were significantly lower than those of both radiologists (all *p* < 0.001).

**Table 1:** Comparison of the diagnostic accuracy between ChatGPT and radiologists

	Correct answer (accuracy rate [%])			
	Final diagnosis	*p* value^*^	Differential diagnosis	*p* value^*^
GPT-4-based ChatGPT	46/106 (43%)		62/106 (58%)	
Reader 1 (Radiology resident)	43/106 (41%)	0.78	61/106 (58%)	0.99
Reader 2 (Board-certified radiologist)	56/106 (53%)	0.22	71/106 (67%)	0.26
GPT-4V-based ChatGPT	9/106 (8%)		15/106 (14%)	
Reader 1 (Radiology resident)	43/106 (41%)	< 0.001**	61/106 (58%)	< 0.001**
Reader 2 (Board-certified radiologist)	56/106 (53%)	< 0.001**	71/106 (67%)	< 0.001**

*ChatGPT* Chat Generative Pre-trained Transformer, *GPT-4* Generative Pre-trained Transformer-4, *GPT-4V* Generative Pre-trained Transformer-4 with vision

*Chi-square tests are performed to compare the accuracy rates between GPT-4-based ChatGPT and each radiologist, as well as between GPT-4V-based ChatGPT and each radiologist

**p < 0.05

### Radiologists’ diagnostic accuracy with ChatGPT’s assistance

Reader 1’s diagnostic accuracy increased from 41% (43/106) to 46% (49/106) for final diagnoses and from 58% (61/106) to 64% (68/106) for differential diagnoses with the assistance of GPT-4-based ChatGPT. Similarly, Reader 2’s diagnostic accuracy increased from 53% (56/106) to 58% (62/106) for final diagnoses and from 67% (71/106) to 73% (77/106) for differential diagnoses with the assistance of GPT-4-based ChatGPT. In contrast, with the assistance of GPT-4V-based ChatGPT, there was no improvement in diagnostic accuracy for either Reader 1 or Reader 2.

### Categorical analysis of ChatGPT’s diagnostic accuracy

Detailed diagnostic accuracy rates for ChatGPT are shown in Table 2. Given the limited number of correct diagnoses by GPT-4V-based ChatGPT, a categorical analysis was considered inappropriate due to the limited statistical power. Thus, we conducted a categorical analysis only for GPT-4-based ChatGPT’s diagnostic accuracy.

**Table 2:** ChatGPT’s diagnostic accuracy categorized by tumor and nontumor groups

	Correct answer (accuracy rate [%])			
	GPT-4-based ChatGPT		GPT-4V-based ChatGPT	
	Final diagnosis	Differential diagnosis	Final diagnosis	Differential diagnosis
Total (*n* = 106)	46/106 (43%)	62/106 (58%)	9/106 (8%)	15/106 (14%)
Tumor group (*n* = 45)	14/45 (31%)	22/45 (49%)	4/45 (9%)	5/45 (11%)
Nontumor group (*n* = 61)	32/61 (52%)	40/61 (66%)	5/61 (8%)	10/61 (16%)
Tumor group (*n* = 45)^a^	14/45 (31%)	22/45 (49%)	4/45 (9%)	5/45 (11%)
Bone tumor (*n* = 24)	8/24 (33%)	14/24 (58%)	2/24 (8%)	3/24 (13%)
Soft tissue tumor (*n* = 22)	6/22 (27%)	9/22 (41%)	2/22 (9%)	2/22 (10%)

*ChatGPT* Chat Generative Pre-trained Transformer, *GPT-4* Generative Pre-trained Transformer-4, *GPT-4V* Generative Pre-trained Transformer-4 with vision

^a^One case presents both a bone tumor and a soft tissue tumor

When comparing the tumor and nontumor groups, the final and differential diagnostic accuracy rates were 31% (14/45) and 49% (22/45) for the tumor group, and 52% (32/61) and 66% (40/61) for the nontumor group, respectively. The tumor group showed significantly lower final diagnostic accuracy rates compared to the nontumor group (*p* = 0.03), while there was no significant difference between the differential diagnostic accuracy rates of the two groups (*p* = 0.11). Within the tumor group, the final and differential diagnostic accuracy rates were 33% (8/24) and 58% (14/24) in bone tumor cases, and 27% (6/22) and 41% (9/22) in soft tissue tumor cases, respectively (one presented both a bone tumor and a soft tissue tumor). When comparing the diagnostic accuracy rates between bone tumor and soft tissue tumor cases, no significant difference was observed in either the final or differential diagnosis (*p* = 0.75 and 0.38, respectively).

The diagnostic accuracy rates for the nontumor etiologies are presented in Table 3. GPT-4-based ChatGPT demonstrated relatively higher final diagnostic accuracy for congenital/developmental abnormality and dysplasia, trauma, and anatomical variant categories. In contrast, its final diagnostic accuracy was relatively lower for arthritis/arthropathy, infection, and metabolic disease categories.

**Table 3:** ChatGPT’s diagnostic accuracy in nontumor etiologies

	Correct answer (accuracy rate [%])			
	GPT-4-based ChatGPT		GPT-4V-based ChatGPT	
	Final diagnosis	Differential diagnosis	Final diagnosis	Differential diagnosis
Muscle/soft tissue/nerve disorder (*n* = 12)	7/12 (58%)	11/12 (92%)	2/12 (17%)	3/12 (25%)
Arthritis/arthropathy (*n* = 10)	4/10 (40%)	4/10 (40%)	1/10 (10%)	1/10 (10%)
Infection (*n* = 8)	3/8 (38%)	5/8 (63%)	0/8 (0%)	1/8 (13%)
Congenital/developmental abnormality and dysplasia (*n* = 6)	4/6 (67%)	4/6 (67%)	0/6 (0%)	0/6 (0%)
Trauma (*n* = 6)	5/6 (83%)	5/6 (83%)	1/6 (17%)	2/6 (33%)
Metabolic disease (*n* = 5)	2/5 (40%)	3/5 (60%)	0/5 (0%)	0/5 (0%)
Anatomical variant (*n* = 4)	3/4 (75%)	3/4 (75%)	0/4 (0%)	0/4 (0%)
Others (*n* = 10)	4/10 (40%)	5/10 (50%)	1/10 (10%)	3/10 (30%)

*ChatGPT* Chat Generative Pre-trained Transformer, *GPT-4* Generative Pre-trained Transformer-4, *GPT-4V* Generative Pre-trained Transformer-4 with vision

## Discussion

This study demonstrated the diagnostic accuracy of GPT-4-based ChatGPT and GPT-4V-based ChatGPT in musculoskeletal radiology. The diagnostic accuracy of GPT-4-based ChatGPT (based on the patient’s medical history and imaging findings) was significantly higher than that of GPT-4V-based ChatGPT (based on the patient’s medical history and images). Regarding the comparison between ChatGPT and radiologists, GPT-4-based ChatGPT’s diagnostic accuracy was comparable to that of a radiology resident but lower than that of a board-certified radiologist. While GPT-4V-based ChatGPT’s diagnostic accuracy was significantly lower than that of both radiologists. The diagnostic accuracy of radiologists improved with GPT-4-based ChatGPT’s assistance, but not with GPT-4V-based ChatGPT’s assistance. In the analysis of GPT-4-based ChatGPT’s diagnostic accuracy per category, GPT-4-based ChatGPT’s final diagnostic accuracy rate was significantly lower for the tumor group compared to the nontumor group. Within the tumor group, the accuracy rates for the final and differential diagnoses were relatively higher for bone tumor cases compared to those of soft tissue tumor cases, although the differences were not significant.

To the best of our knowledge, this study is the first in the field of musculoskeletal radiology to investigate the diagnostic capability of GPT-4 and GPT-4V-based ChatGPTs and to compare these to radiologists’ performance. Although a previous study has reported that GPT-3-based ChatGPT can generate coherent research articles in musculoskeletal radiology [20], no study has evaluated the diagnostic performance of GPT-4 and GPT-4V-based ChatGPTs in this field. This study provides valuable insights into the strengths and limitations of using ChatGPT as a diagnostic tool in musculoskeletal radiology.

While ChatGPT holds promise as a useful tool in musculoskeletal radiology, radiologists should recognize its capabilities and exercise caution when incorporating ChatGPT into clinical practice. This study demonstrated that the diagnostic accuracy of GPT-4-based ChatGPT was significantly higher than that of GPT-4V-based ChatGPT. These results indicated that the GPT-4V-based ChatGPT’s capability to process images and extract imaging findings is insufficient. A recent study has reported that GPT-4V-based ChatGPT exhibited limited interpretive accuracy in analyzing radiological images [25]. One factor contributing to the underperformance of GPT-4V-based ChatGPT was perhaps its insufficient training in medical images. In OpenAI’s statements, they considered the current GPT-4V to be unsuitable for performing the interpretation of medical images and replacing professional medical diagnoses due to inconsistencies [5]. For further improvements of GPT-4V-based ChatGPT’s diagnostic accuracy, exploring techniques such as retrieval-augmented generation, fine-tuning with reinforcement learning from human feedback, and training vision models on a wide range of medical images should be considered [26]. Since textual information is the only feasible support option to date, providing the appropriate description of imaging findings is crucial when utilizing ChatGPT as a diagnostic tool in clinical practice. Regarding the comparison between ChatGPT and radiologists, GPT-4V-based ChatGPT’s diagnostic performance was significantly lower than that of radiologists, and GPT-4-based ChatGPT’s diagnostic performance was comparable to that of radiology residents but did not reach the performance level of board-certified radiologists. ChatGPT may assist radiologists in the diagnostic process; however, ChatGPT alone cannot fully replace the expertise of radiologists and should only be used as an adjunct tool.

Although GPT-4-based ChatGPT alone cannot replace the expertise of radiologists, it is capable of enhancing diagnostic accuracy and assisting radiologists in narrowing down differential diagnoses as part of the diagnostic workflow in musculoskeletal radiology. Furthermore, ChatGPT has been shown to provide valuable assistance to radiologists in various tasks, including supporting decision-making, determining imaging protocols, generating radiology reports, offering patient education, and writing medical publications [26, 27]. The implementation of ChatGPT into radiological practices has the potential to optimize the diagnostic process, resulting in time savings and a decreased workload for radiologists, thereby increasing overall efficiency.

This study also revealed that the diagnostic accuracy of GPT-4-based ChatGPT may vary depending on the etiology of the disease; it was significantly lower in the tumor group compared to the nontumor group. This lower diagnostic accuracy in neoplastic diseases could be attributed to the challenging nature of interpreting complex cases, due to the wide variety of histopathological types and imaging findings [23, 28]. Rare neoplastic diseases may be more challenging for ChatGPT due to the limited literature and a lack of established typical imaging findings. Although no significant difference in diagnostic accuracy rates was observed between bone tumor and soft tissue tumor cases, bone tumor cases showed relatively higher accuracy rates compared to soft tissue tumor cases. While soft tissue tumors of both benign and malignant nature often share overlapping imaging features [29], bone tumors have grading systems that allow for the assessment of malignancy risk based on their growth patterns [30, 31]. This distinction may be one of the contributing factors to the relatively higher differential diagnostic accuracy for bone tumors compared to soft tissue tumors. On the other hand, the significantly higher accuracy rates for the final diagnosis of the nontumor group indicated that GPT-4-based ChatGPT may be particularly useful in diagnosing non-neoplastic diseases in musculoskeletal radiology. Among non-neoplastic diseases, cases of congenital/developmental abnormality and dysplasia, traumatic disease, and anatomical variants showed relatively higher final diagnostic accuracy. These relatively higher accuracies may be attributed to characteristic keywords in patient’s medical history and imaging findings for these conditions.

This study had several limitations. First, ChatGPT’s performance in generating diagnoses was conducted in the controlled environment of the “Test Yourself” cases, which may not fully represent the broader range of musculoskeletal radiology cases. This selection bias could affect the generalizability of the results and may not capture the full spectrum of diagnostic challenges encountered in real-world clinical practice. Second, the “Test Yourself” cases represent a potential for bias since these cases may have been included in the training data of ChatGPT. This bias may lead to an overestimation of ChatGPT’s diagnostic accuracy. Third, this study utilized the descriptions of imaging findings provided by authors aware of the final diagnosis in the “Test Yourself” cases. This may have introduced a bias, which could lead to an overestimation of GPT-4-based ChatGPT’s diagnostic accuracy. Further studies are necessary to mitigate this bias, including evaluating ChatGPT’s diagnostic accuracy utilizing the descriptions of imaging findings provided by radiologists blinded to the final diagnosis. Fourth, radiologists’ diagnoses with the assistance of ChatGPT may introduce a bias, potentially leading to an overestimation of ChatGPT’s capabilities as a diagnostic support tool. Fifth, this study did not conduct a categorical analysis for GPT-4V-based ChatGPT’s diagnostic accuracy due to the limited number of correct diagnoses, which limits the statistical power of the analyses. Sixth, this study did not perform a statistical analysis for ChatGPT’s diagnostic accuracy in non-neoplastic etiologies due to the limited number of cases. Finally, this study did not investigate hallucinations, a critical limitation of large language models [25, 26, 32]. Radiologists need to be aware of hallucinations when utilizing ChatGPT as a diagnostic tool in clinical practice. Further studies are necessary to explore the characteristics and mitigation strategies of hallucinations for optimal utilization of ChatGPT.

In conclusion, this study evaluated the diagnostic accuracy of both GPT-4-based ChatGPT and GPT-4V-based ChatGPT in musculoskeletal radiology. When GPT-4-based ChatGPT utilized the descriptions of imaging findings provided by distinguished radiologists, its diagnostic performance was comparable to that of radiology residents but did not reach the performance level of board-certified radiologists. In contrast, GPT-4V-based ChatGPT, which independently evaluates imaging findings, showed poor diagnostic ability. Since textual information is the only feasible support option to date, providing the appropriate description of imaging findings is crucial when utilizing ChatGPT as a diagnostic tool in clinical practice. While ChatGPT may assist radiologists in narrowing down the differential diagnosis and improving the diagnostic workflow, radiologists need to be aware of its capabilities and limitations for optimal utilization.

## Abbreviations

ChatGPT: Chat Generative Pre-trained Transformer
GPT-4: Generative Pre-trained Transformer-4
GPT-4V: Generative Pre-trained Transformer-4 with vision
